# Development of Iron-Chelating/Antioxidant Nutraceuticals and Natural Products as Pharmaceuticals for Clinical Use in Diseases with Free Radical Pathologies

**DOI:** 10.3390/nu17203270

**Published:** 2025-10-17

**Authors:** George J. Kontoghiorghes

**Affiliations:** Postgraduate Research Institute of Science, Technology, Environment and Medicine, Limassol 3021, Cyprus; kontoghiorghes.g.j@pri.ac.cy; Tel.: +357-26272076

**Keywords:** nutraceutical repurposing, iron chelating, antioxidant nutraceuticals, ascorbic acid, quercetin, curcumin, fisetin, maltol, lipoic acid, deferiprone, deferoxamine

## Abstract

Antioxidant activity is a normal physiological function that is essential for healthy living, and it is maintained by antioxidant dietary nutrients. However, increases in free radical production and oxidative toxicity in many clinical conditions can cause serious and sometimes irreversible damage. Despite many investigations, including hundreds of clinical trials suggesting that there are health benefits obtained from the use of natural antioxidants, no antioxidant drugs have yet been developed for the treatment of any disease associated with free radical pathology. Millions of people choose to use nutraceutical and natural product antioxidants as therapeutics and also for chemoprevention against cancer and other diseases. New academic efforts and strategies are required for the development of antioxidant drugs in clinical practice in the absence of interest by the pharmaceutical and nutraceutical industries. One of the most effective antioxidant therapeutic strategies is inhibition by chelators of iron involved in the catalytic formation of free radical reactions and their associated damage. Hundreds of phytochelators have been shown to inhibit oxidative damage, similar to the iron-chelating drugs deferiprone and deferoxamine. In particular, several nutraceuticals and natural products such as ascorbic acid, quercetin, curcumin, fisetin, lipoic acid, and maltol have been shown to have high antioxidant activity and iron-binding capacity, as well as other effects on iron metabolism, in pre-clinical studies and clinical trials involving different categories of patients. For example, ascorbic acid and maltol–iron complexes are sold as pharmaceutical products for the treatment of iron deficiency. The development of nutraceuticals as antioxidant drugs may involve one or more applications, such as short- or long-term treatments, single-drug or combination therapies, and also different targets, such as the prevention, treatment, or post-treatment of diseases associated with free radical pathology as well as ferroptosis. The academic efforts surrounding the developments of iron-chelating nutraceuticals or natural products into antioxidant pharmaceuticals should fulfill all of the regulatory requirements and include clinical tests of antioxidants in rare or untreatable diseases, as well as the involvement of government translational research institutions and expert groups that specialize in regulatory drug affairs, among others.

## 1. Introduction

There is a major need for the development and introduction of antioxidant pharmaceuticals in medicine. Millions of pre-clinical studies and hundreds of clinical trials have been carried out so far on potential antioxidant drugs, nutraceuticals, and natural products with possible uses in clinical practice in relation to diseases of free radical (FR) pathology. Despite all of these pre-clinical and clinical experimental efforts, no antioxidant pharmaceuticals have been developed so far for clinical use, mainly as a result of insufficient and unconvincing data being submitted for regulatory drug authority approval, and also for commercial or other reasons [1,2,3,4,5,6].

In the meantime, millions of people are regularly using traditional folk medicines, including natural products and nutraceuticals with antioxidant properties, without a physician’s prescription [1,2,3,4,7]. Despite the current wide use of nutraceutical antioxidants being based mostly on chemoprevention for diseases associated with increased oxidative stress and redox imbalance, their clinical development and potential application as therapeutics could benefit millions of patients [5,6,8,9]. In this context, the maintenance of redox homeostasis, including the development of antioxidant pharmaceuticals, is important not only for the prevention of serious diseases and for healthy living, but also potentially for the treatment of many diseases that are associated with FR pathology, including diseases where effective treatments are not available, such as cancer and neurodegenerative diseases [5,6,8,9].

Under normal physiological conditions, many FRs, such as hydroxyl radical, superoxide, and nitric oxide, as well as other reactive oxygen species (ROS) such as hydrogen peroxide and lipid peroxides, participate in many normal physiological functions, including the metabolism of natural compounds, drugs, and other xenobiotic molecules, the oxidation of food products, and cell signaling and aging [10,11,12,13,14,15]. However, in many diseases, changes around, for example, nutrient homeostasis, including iron or copper overload, or changes in other endogenous factors such as mitochondria malfunction, and also exogenous factors such as heavy metals or UV irradiation, can all cause an increase in FR and ROS production and related cascades, leading to redox homeostatic imbalance and also oxidative toxicity [16,17,18,19,20,21]. This form of toxicity progressively evolves into oxidative stress, which can be damaging to the cells affected unless it can be reversed by an effective innate antioxidant system and the damage to the cells is also restored by a repair system [5,10,11,22,23,24,25,26,27]. The reversal of oxidative stress by the antioxidant system is generally accomplished by an antioxidant process involving the prevention, delay, or neutralization of the harmful effects of the increased production of FR and ROS [5,22,23,24,25,26,27].

The antioxidant system under normal physiological conditions is composed of different mechanisms and processes that mainly involve antioxidant enzymes and antioxidant molecules, which are used to maintain redox homeostasis. In this context, several antioxidant enzymes are involved, including superoxide dismutase, glutathione peroxidase, and catalase. Similarly, natural antioxidant molecules are also involved, including endogenous glutathione, exogenous dietary molecules such as Vitamin C and E, and also many other nutrients such as polyphenols [5,22,23,24,25,26,27,28].

In some cases, the antioxidant defense system could become insufficient or ineffective, and this could lead to oxidative stress toxicity (OST). This form of toxicity can cause serious oxidative damage to biomolecules, such as the modification of DNA, sugars, lipids, and proteins. Furthermore, persistent oxidation-related damage could also progressively lead to structural and functional damage to organelles, cells, tissues, and organs [5,26,29,30,31,32].

One of the major factors associated with oxidative damage is related to redox iron-ion toxicity [8,23,26]. Iron is an essential transition metal ion, playing an important catalytic role in the production of FRs in biological systems, including participation in the metalloproteins of mitochondria, metalloenzymes, or in the form of low-molecular-weight iron complexes involved in Fenton reactions [23,26,33,34]. In this context, iron plays an essential role in the maintenance of redox homeostasis. Similarly, iron is also an essential component of many proteins involved in many other physiological activities and functions, such as oxygen storage by myoglobin and oxygen transport by hemoglobin [33,34,35]. Most importantly, the recently discovered essential roles of iron in the peroxidation of lipids in cell membranes and other compartments in ferroptosis, which is a programmed cell death process identified in almost all diseases, including all types and stages of cancer, highlights, in general, the critical role played by iron and iron metabolism in general, in both the normal and disease states [36,37,38,39,40,41,42].

In normal physiological conditions, the iron metabolism is strictly controlled and regulated by specific proteins at all the stages of iron absorption, transport, storage, and utilization, without redox or other toxicity implications [33,34,35]. For example, the iron transport protein transferrin in blood can mobilize and carry up to two molecules of iron, which can then be delivered to all cells in the body via transferrin receptors [33,34,35,43]. Similar iron-binding activity is also shown by the transferrin sister protein lactoferrin, which is found in bodily secretions and neutrophils [44,45]. Both of these proteins are regarded as powerful natural iron chelators/antioxidants because they mobilize labile iron, which is potentially toxic. These are also involved in antimicrobial and many other physiological activities [34,35,43,44,45]. Similarly, the iron-storage protein ferritin, which is found in all cells in the body and can store up to 4500 molecules of iron in the form of polynuclear oxohydroxy iron complexes, is not normally involved in redox reactions [34,35,46,47]. However, ferritin damage can cause the release of labile iron, which can become redox active and potentially toxic. In particular, ferritinophagy is observed during ferroptosis, which can cause the release of toxic labile iron and lead to the increased production of FRs, ROS, and, subsequently, to programmed cell death [5,36]. In this context, natural and synthetic chelators, which can mobilize labile iron, may potentially inhibit iron-catalyzed FRs and ROS and the associated processes of OST and damage, as well as ferroptosis [5,26,34,36]. Other antioxidants such as Vitamin E can also minimize OST and inhibit ferroptosis, but cannot inhibit the initiation of FRs, ROS, and related cascades catalyzed by iron [26,34,36].

Different ligands and chelators are involved in the binding of iron in biological systems, which, in almost all cases, contain the electron-donating atoms oxygen, nitrogen, or sulfur for bond formation with iron. In particular, chelators must possess at least two ligands, which can form a ring structure with iron or another metal ion as the closing member of the ring. In this context, the affinity of various chelators for iron is different and depends on the structural and molecular characteristics of the chelator. Furthermore, the same structural characteristics determine the other properties of the chelators, including toxicological, physicochemical, pharmacological, and other parameters [26,34,47]. Similarly, the functional effects of iron chelators are different, including the activation or inhibition of redox-active iron participating in Fenton reactions, as well as of the other iron catalytic centers associated with FR pathologies, in many clinical conditions and many diseases associated with ferroptosis [5,34,47].

Iron and iron chelators play very important clinical, biological, and redox roles in normal physiological conditions, as well as in pathological conditions [34,47]. In this context, the design and development of effective and target-specific iron-chelating/antioxidant drugs could have a major impact on the treatment of many diseases associated with FR pathology [5]. Similarly, the design of target-specific iron-chelating/antioxidant drugs could play a pivotal role in the regulation or modulation of ferroptosis and all associated diseases. In this context, it has been suggested that many naturally occurring polyphenols and other classes of phytochelators, including many synthetic nutraceuticals, can play a major role in the development of iron chelators/antioxidants and in the treatment of many diseases associated with FR pathologies, as well as in conditions of increased oxidative damage and many diseases associated with ferroptosis [1,2,3,4,5,26,28].

The main purpose of this review is to introduce new approaches and strategies for the development of a new class of pharmaceuticals using iron-chelating nutraceuticals or other natural compounds with antioxidant properties for the treatment of various diseases associated with FR pathologies and ferroptosis.

## 2. The Development of Nutraceuticals for Use in Antioxidant Pharmaceuticals

The design and development of new drugs is a time-consuming, tedious, and very expensive process, which in most cases is undertaken by pharmaceutical companies and is based on commercial criteria [1,2,5,6,48,49]. In all of these cases, robust therapeutic, diagnostic, safety, and other criteria are required by drug regulatory authorities for the approval of a new drug. Furthermore, sufficient evidence and appropriate information is required regarding the improved efficacy, safety, and other advantages of the proposed candidate drug over any other approved drugs for the treatment of a specific disease [49]. Such information includes a significant amount of experimental data from chemical synthesis and chemical properties, and also from pre-clinical studies, toxicology, and clinical trials [5,49].

No antioxidant drugs are yet available in clinical practice. However, there are many indications, including thousands of pre-clinical studies and hundreds of clinical trials, suggesting that the development of antioxidant drugs could play an important role not only in chemoprevention, but also in the treatment of many diseases [1,2,3,4,5,6,28]. Despite all these indications of the possible health and therapeutic benefits that can be obtained from the use of antioxidant natural products and nutraceuticals, there appears to be insufficient interest by both the pharmaceutical and nutraceutical industries for undertaking such developmental projects, mainly because of commercial and proprietary considerations [49]. In this context, new approaches and strategies are required by the academic community for the development of antioxidant drugs, including the clinical development of related nutraceuticals and natural products for applications in medicine [5,6,49].

There are several advantages that motivate the general strategy regarding the development of nutraceuticals and natural products for use as antioxidant drugs. These include the availability of the supporting published scientific literature, the wide public daily use, as well as the general awareness of the potential health benefits of nutraceutical and natural products antioxidants in everyday life [1,2,3,4,28]. Similarly, additional advantages in their developmental process include, for instance, the presence of prior extensive human use and the knowledge and experience surrounding the posology and safety parameters of different pharmaceutical-grade nutraceutical formulations for oral or parenteral administration [1,2,3,4,28,49].

The developmental process for transforming nutraceuticals and other natural products into antioxidant pharmaceuticals could involve several approaches in relation to the specificity of the targeting methods, such as mechanisms and pathways of antioxidant activity, and also the selection of specific diseases associated with FR pathologies [5,6,26]. In particular, the identification of a specific target related to increased FR/ROS production and OST, as well as the selection of an appropriate antioxidant with effective posology, will be critical for determining the efficacy of the therapeutic effects of the antioxidant process [5,6,50]. The targeting options in each disease associated with FR pathologies may also involve one or more aspects of increased production of FR/ROS and OST damage—for example, one or more organ(s), specific cell type(s), sub-cellular compartment(s), metabolic pathway(s), and therapeutic procedures [5,6]. Furthermore, the targeting options for potential antioxidant pharmaceuticals are likely to increase in the future, considering that almost all diseases have been associated with ferroptosis, which can be inhibited or modulated by both antioxidants and chelators [5,36,37,38,39,40,41,42].

The process for the selection and development of nutraceuticals and other natural products for antioxidant therapies could also be simplified with regard to regulatory authority requirements by selecting diseases with ineffective therapies, such as different types of cancer, neurodegenerative and other similar conditions, ischemia–reperfusion injury, drug toxicity, or renal, cardiac, liver, and other organ damage, etc. [5,49]. In such cases, more relaxed procedures and requirements for drug development are considered by the regulatory drug authorities based on the context of a risk/benefit assessment, similar to those applied for orphan drugs, emergency drugs, and generic drug repurposing [5,49]. In general, most regulatory requirements for the development of nutraceuticals and natural products for use in pharmaceuticals are similar for all drug categories, regardless of the developmental procedure. However, the critical point in each new drug case is that a significant improvement or remission without serious toxicity should be shown in patients in a disease category by the proposed antioxidant drug in comparison to placebo by using a sufficient number of patients in double-blind randomized controlled clinical trials [5,49].

## 3. Limitations of the Use of Iron-Chelating Drugs as Antioxidants for Clinical Use

One of the most promising approaches for identifying antioxidants for clinical use is the repurposing of effective iron-chelating drugs, which, in principle, can inhibit the catalytic activity of iron related to the Fenton reaction in diseases associated with FR pathologies and diseases associated with ferroptosis [5,6,36,37,38,39,40,41,42]. Although many natural and synthetic chelators can potentially inhibit this form of redox iron toxicity, existing iron-chelating drugs appear to be the most advanced candidates, in drug-regulatory terms, for repurposing and development as antioxidant pharmaceuticals for clinical use [5,6,26].

**Figure 1:**
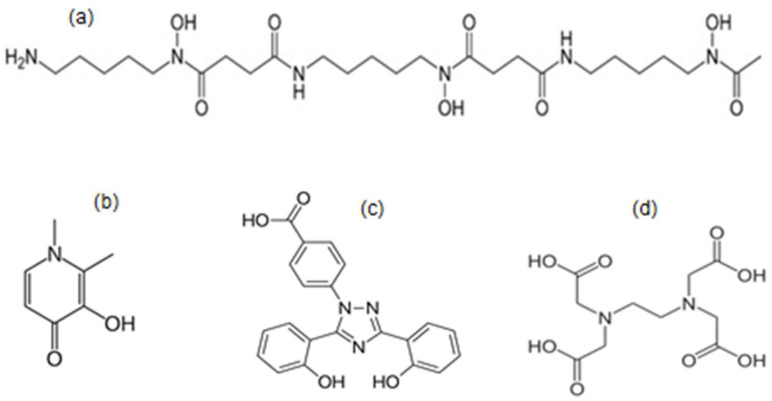
The chemical structure of the iron-chelating drugs deferoxamine, deferiprone and deferasirox, which are widely used for the treatment of transfusional iron overload and also of the heavy metal chelating drug EDTA, which is widely used in alternative medicine clinics. Deferoxamine (**a**) is administered subcutaneously and intravenously, deferiprone (**b**) and deferasirox (**c**) orally and EDTA (**d**) intravenously. (EDTA: ethylene diaminetetraacetic acid).

There are three regulatory-approved iron-chelating drugs, namely, deferoxamine (DF), deferiprone (L1), and deferasirox (DFRA), which are widely used for the treatment of transfusional iron overload in thalassaemia and other similar conditions (Figure 1) [51]. Thousands of iron-loaded patients are using these life-saving drugs on a daily basis for the elimination of excess iron, which accumulates in the heart and other organs following regular red blood cell transfusions [52,53,54]. In contrast, high morbidity and mortality rates are observed in thalassaemia patients in developing countries, who receive regular red blood cell transfusions but cannot afford the cost of chelation therapy for the elimination of excess iron from the body. In such cases, the major cause of mortality, which usually occurs by the age of 20 years, is congestive cardiac failure associated with cardiac iron overload toxicity [55,56]. In contrast to the high mortality rate observed in non-chelated regularly transfused thalassaemia patients, the use of L1/DF in combination, or, in some cases, L1 monotherapy, appears to cause an increase in the life expectancy and reduction in the morbidity and mortality rates of thalassaemia patients to levels of survival approaching those of normal individuals [57,58,59,60,61].

The general properties of L1, DF, and DFRA, including iron-binding, chemical, pharmacological, toxicological, and clinical effects, have been previously reviewed (Figure 1) [26,34,51]. The recommended doses used for each of the chelating drugs in iron-loaded thalassaemia patients is 40–60 mg/kg/day for subcutaneous or intravenous DF, 75–100 mg/kg/day for oral L1, and 20–40 mg/kg/day for oral DFRA. Overall, there are variable modes of action, organ targeting, and efficacy in the removal of iron and also of toxicity among these three chelating drugs in iron-loaded thalassaemia patients [26,34,51]. However, despite the wide use of L1, DF, and DFRA in thalassaemia and other diseases related to iron overload, there are limitations around their use in non-iron-loading diseases due to serious toxic side-effects observed mainly by DF and DFRA in non-iron-loaded patients [5,62,63,64]. In this context, the clinical use of both DF and DFRA in non-iron-loaded patients is restricted due to toxicity implications [65,66,67,68,69,70,71]. In particular, the administration of both DF and DFRA is not encouraged in non-iron-loaded patients, as well as in regularly transfused thalassaemia patients with serum ferritin lower than 0.5 mg/L [63,64].

In contrast to DF and DFRA, many clinical trials have been carried out using L1 in different non-iron-loaded conditions with encouraging therapeutic outcomes, including antioxidant effects, especially when OST parameters were monitored [72,73,74,75,76,77]. However, several toxic side-effects have also been reported during the use of L1 in iron-loaded thalassaemia patients, as well as in patients with normal iron stores. The most serious toxic side-effects of L1 are agranulocytosis (1%>) and neutropenia (5%>), which are reversible [51,78,79]. In this context, weekly or fortnightly mandatory blood count monitoring is recommended for prophylaxis for all patients using L1. Other less serious toxic side-effects caused by L1 include gastric intolerance, joint/musculoskeletal pains, and zinc deficiency [80,81,82,83,84].

Overall, L1 appears to be the only iron-chelating drug that is not restricted for use in patients with normal iron stores and that has potential use as a chelator/antioxidant drug in diseases of FR pathology [5,6]. However, as discussed above, there are also disadvantages to its long-term therapeutic use, including the prospect of toxic side-effects in a small proportion of patients, and also the need for regular mandatory blood counts for prophylaxis [78,79].

It is anticipated that the identification and development of naturally occurring iron chelators/antioxidants, including related nutraceuticals and other natural compounds, could possibly provide some advantages over synthetic iron-chelating/antioxidant drugs. In particular, it is anticipated that some of the iron-chelating/antioxidant nutraceuticals involved in clinical testing may be at an advanced stage of development for clinical use, and that, overall, they will be more effective and less toxic than L1, DF, and DFRA for use in some diseases associated with FR pathologies or ferroptosis.

## 4. Repurposing of Selected Nutraceuticals as Iron-Chelating Antioxidant Drugs in Medicine

Nutraceuticals are considered to be dietary food product supplements, mainly of plant origin, with potential pharmaceutical properties, which may be used for the prevention of diseases or the facilitation of the treatment of diseases [1,2,3,4,85]. Nutraceuticals are not pharmaceutical products or drugs, are not prescribed for clinical purposes, and are also not extensively tested or regulated by the drug regulatory authorities. Many natural plant products are isolated and sold as nutraceuticals, especially as traditional folk medicines in developing countries, where much lower prices are generally paid in comparison to synthetic registered drugs used for the same clinical condition [7,28,48,49]. In general, nutraceutical products can be isolated from plants or, in most cases, synthesized chemically, sold in different formulations, and advertised as offering general health and medical benefits, but not prescribed by physicians for the treatment of any medical condition [1,2,3,4,7,28].

The natural origin and the long-term use of natural products or synthetic nutraceuticals in folk medicine have increased their acceptability “as safe products” for consumption in the general public, as well as increasing their prospects for drug development and use in medicine [85]. As a result, many nutraceuticals have been tested in pre-clinical and clinical studies in different diseases and, in some cases, in the context of antioxidant therapies as monotherapies and also in combination with other drugs for the treatment of diseases associated with FR pathologies [1,2,3,4,5,25,26,85,86,87,88,89,90,91].

Many natural plant products, including nutrients and different nutraceuticals that are known to have antioxidant properties, also appear to have metal-chelating sites with iron-binding potential [26,86,87,88,89,90,91]. Some of these iron phytochelators include mimosine, tropolone, maltol, emodin, gentisic acid, quercetin, curcumin, fisetin, lipoic acid, and many others [26]. Furthermore, many of these express redox activity through their iron complexes, including potent antioxidant properties in relation to the inhibition of the Fenton reaction [26,76]. In this context, the identification of natural products and nutraceuticals with iron-chelating/antioxidant properties may increase the prospects of their development as iron-chelating/antioxidant pharmaceuticals [26,76,86,87,88,89,90,91].

**Figure 2:**
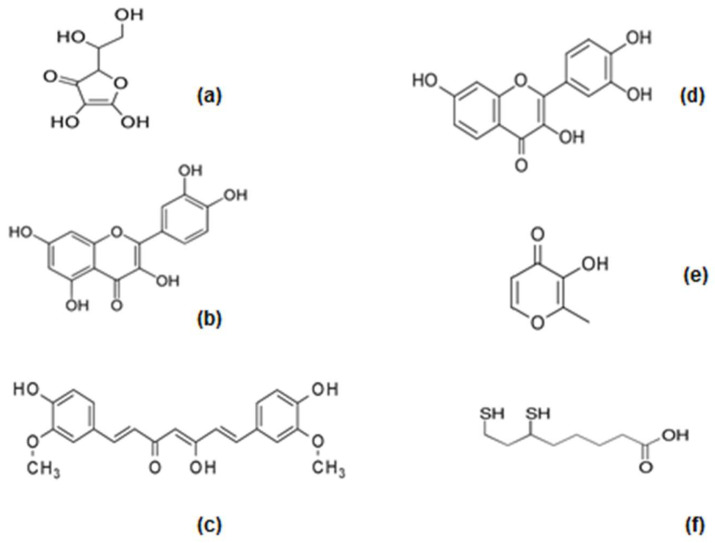
The chemical structure of iron-chelating/antioxidant nutraceuticals and other natural products used in clinical trials in different categories of patients: ascorbic acid (**a**), quercetin (**b**), curcumin (enol form) (**c**), fisetin (**d**), maltol (**e**), and dihydrolipoic acid (**f**). The iron-binding sites include two adjacent hydroxyl groups and/or alpha keto hydroxy groups in ascorbic acid, quercetin, and fisetin, which are similar to catechol and maltol, respectively. Similarly, a beta keto enol iron-binding site is present in curcumin, and a di-thiol iron-binding site is present in dihydrolipoic acid.

Among the many and various groups of antioxidant nutraceuticals and natural products, there is a large category of available compounds with iron-chelating/antioxidant potential that have not yet been fully characterized for their iron-binding capacity, redox, and other interactions with iron and other metals. In particular, some of the most widely known and clinically used or tested antioxidant nutraceuticals and natural products belong to this category, which includes ascorbic acid (or Vitamin C), quercetin, curcumin, fisetin, maltol, and lipoic acid (Figure 2) [26,76,86,87,88,89,90,91].

### 4.1. Antioxidant and Other Therapeutic Effects of Ascorbic Acid and Its Interactions with Iron

Ascorbic acid is an essential nutrient and important vitamin used for the normal growth and development of humans, while its deficiency can result in serious diseases. Ascorbic acid is generally used in clinical practice for the treatment of scurvy, hypovitaminosis C, and similar conditions of Vitamin C deficiency, as well as in combinations with several other drugs for the treatment of other conditions [91,92,93,94]. Ascorbic acid can be found in the World Health Organization’s list of essential medicines, and is the most widely used antioxidant and clinically tested nutraceutical for different diseases. It is also regarded as the safest and most effective drug needed for the maintenance of healthy living (Figure 2) [95].

**Figure 3:**
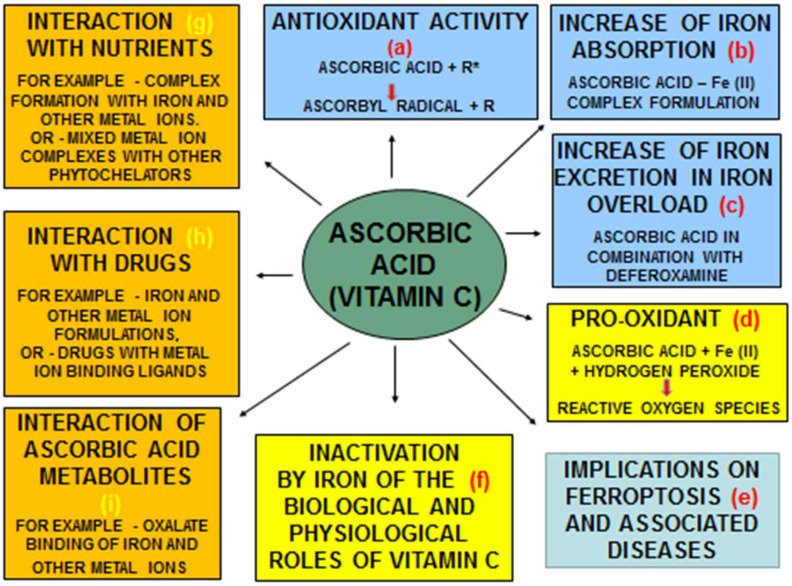
A diagram showing some of the biological, physiological, and pharmacological effects of ascorbic acid (Vitamin C) on iron and iron metabolism and vice versa. The antioxidant activity of ascorbic acid is based on its reaction and neutralization of free radicals (**a**). The ascorbic acid iron (II) complex formulation is used as a drug supplement for the treatment of iron deficiency (**b**). Ascorbic acid is used in combination with deferoxamine for the elimination of excess iron in the treatment of iron overload (**c**). Ascorbic acid can act as a pro-oxidant in the presence of iron (II) and hydrogen peroxide, causing increasing production of reactive oxygen species (**d**). Ascorbic acid can modulate and have different implications on diseases associated with ferroptosis (**e**). The biological and physiological roles of Vitamin C can be inactivated in the presence of iron (**f**). Both iron and ascorbic acid are present in meals and may interact and form complexes. The same applies to other metal ions and phytochelators that are mainly present in fruit and vegetables (**g**). Iron and other metal ions can interact with many drugs possessing ligand or chelating sites. Similarly, ascorbic acid can interact with iron and other metal-ion-containing formulations (**h**). The same interactions of ascorbic acid with iron and other metal ions can also be observed with oxalate and other ascorbic acid metabolites (**i**). (R*: Radical species. R: Non-radical species).

The antioxidant properties and other effects of ascorbic acid, including redox, chelation, therapeutic, pharmacological effects, and toxicological implications, have been previously reviewed [95]. Overall, the general effects of ascorbic acid are modulated by other nutrients and biomolecules, including metal ions. In particular, the interactions between ascorbic acid and iron are of nutritional, physiological, pharmacological, and toxicological interest, with major implications for health and disease [95,96]. Its interactions with iron can take place in many different cells and organs, especially considering that these two essential nutrients are present and are absorbed almost daily from food in the gastrointestinal tract [95,97]. In addition, millions of people are using different pharmaceutical and nutraceutical preparations of these two nutrients daily whilst being unaware of the effects of their interactions and overall implications on their health (Figure 3) [95,96,97,98,99].

It should also be considered that ascorbic acid and its metabolites, including the ascorbate anion and oxalate, have metal-binding capacity and bind iron as well as other metal ions [95]. Similarly, the antioxidant effects and the biological roles of ascorbic acid as a vitamin are generally affected by iron and other metal-ion-complex formations (Figure 3). In particular, ascorbic acid forms a complex with iron (III), followed by reduction to iron (II), which appears to potentiate FR production via the Fenton reaction [100]. The biological and clinical activities of ascorbic acid and the ascorbate–iron complex can also be affected by many other nutrients, including other metal ions, and also different pharmaceutical preparations, including those with iron-binding capacities, such as doxorubicin and other anthracyclines, tetracyclines, hydroxyurea, etc. (Figure 3) [95,101,102,103,104].

One of the main functions and uses of ascorbic acid in nutraceutical formulations is for its antioxidant activity against ROS, which has been implicated in many diseases with FR pathologies, including cancer and aging (Figure 3). In this context, the antioxidant effects of ascorbic acid have been used in many clinical trials involving iron-metabolic diseases, severe pneumonia, severe acute respiratory failure, multiple myeloma, metastatic colorectal carcinoma, metastatic melanoma, coronary artery disease, myocardial infarction, diabetes, dementia, Alzheimer’s disease, sepsis, and many other diseases [91,95,105,106,107,108,109,110,111,112,113].

Some of the advantages of the selective use of ascorbic acid seen in many clinical trials are its high tolerability and safety at high doses. In this context, a wide range of high oral and intravenous doses of ascorbic acid have been used in clinical trials that mainly involve different categories of cancer patients and normal volunteers [114,115,116,117,118,119,120,121]. For example, in a pharmacokinetic 4-week study on patients with metastatic prostate cancer, a range of daily doses from 5 to 60 g of ascorbic acid were administered intravenously. Ascorbic acid was well tolerated by the patients, and a peak plasma concentration of 20.3 mM at the highest dose was observed, with an elimination half-life of about 2 h at different ranges of doses [122]. Similarly, no major toxicity was reported in another study using different dosing protocols of doses of as much as 10 g/day intravenously for 10 days and thereafter 10 g/day orally for several months [121].

The pharmacokinetic and pharmacodynamics characteristics of ascorbic acid and other drugs or nutraceuticals are very important for the design of effective, non-toxic dose protocols for therapeutic use. In this context, several pharmacodynamic studies have been carried out using different doses of ascorbic acid. For example, in one study involving normal volunteers, the oral administration of different single doses of ascorbic acid suggested that the bioavailability was complete at 200 mg, but declined at 500 mg or higher ascorbic acid doses. Furthermore, increased excretion of ascorbic acid and its metabolites oxalate and uric acid was also observed at 1000 mg doses compared to lower doses [123]. The distribution of ascorbic acid was also studied, and, at doses of 200 mg and above, the plasma concentration was estimated to be about 0.075 mM, while in cells of the immune system, it was much higher (neutrophils 1.3 mM, lymphocytes 3.2 mM and monocytes 3.5 mM). A decrease in serum ferritin, which generally reflects the level of bodily iron stores, was also observed during clinical investigations involving ascorbic acid [123]. Further information on the absorption and distribution of ascorbic acid was obtained in another study, where it was found that ascorbic acid is absorbed in the small intestine and can also cross the blood–brain barrier. Furthermore, the variable distribution of ascorbic acid in organs was observed, with the highest concentration reported to be present in the adrenal glands (550 mg/kg), brain (140 mg/kg), and liver (125 mg/kg) [124].

The interactions between ascorbic acid and iron and other metal ions may also have physiological and clinical implications in relation to diseases associated with FR pathologies and ferroptosis (Figure 3) [125,126,127,128,129,130]. For example, under certain conditions, ascorbic acid can reduce ferric to ferrous iron and can act as a pro-oxidant by increasing FR and ROS production [76,95,100]. Similarly, ascorbic acid can also act as a chelator, forming iron complexes or mixed iron complexes with other chelators [76,95,100].

The diverse properties and effects of ascorbic acid can also be used in many clinical conditions, including both the treatment of iron-deficiency-induced anemia and also the treatment of iron overload in combination with DF. In this context, there are many iron formulations used by patients following prescription by physicians or bought over pharmacy counters without prescription for the treatment of iron deficiency, with each formulation claiming high efficacy and low toxicity. One of these formulations is oral ferrous ascorbic acid, which appears to be widely used for the treatment of iron-deficiency-induced anemia in developing countries such as India [131,132,133] (Figure 3). For example, in comparative clinical studies using different iron formulations, it has been shown that the administration of oral ferrous ascorbic acid at doses of 3–6 mg/kg/day for 12 weeks in iron-deficient children has led to substantial increases in hemoglobin levels of about 4–5 g/dL. The results suggest that oral ferrous ascorbic acid at these doses is more efficient than other oral iron formulations such as iron polymaltose and ferrous sulfate [131,132,133] (Figure 3).

Ascorbic acid is also widely used as a standard adjuvant chelating drug therapy, mainly in combination with subcutaneous DF, for the elimination of excess iron in the treatment of transfusional iron overload in thalassaemia (Figure 3) [134,135,136,137,138]. In particular, ascorbic acid levels are considered a major factor influencing the route and level of iron excretion caused in thalassaemia patients during its combination therapy with subcutaneous DF [134,135,136,137,138]. Similarly, many-fold increases in iron excretion were also caused in other categories of iron-loaded patients in addition to thalassaemia patients using the combination of subcutaneous DF and oral ascorbic acid [135,138]. In general, different combination protocols may be considered in such therapies, but, in most cases of iron-loaded thalassaemia patients, the oral administration of ascorbic acid of 200 mg before and 200 mg during the infusion of subcutaneous DF is widely used (Figure 3).

In addition to iron-overload and iron-deficiency diseases, different nutraceutical formulations and posology of ascorbic acid are also widely used daily by the general public as a powerful antioxidant for chemoprevention in relation to cancer, as well as infectious or other diseases. In particular, the anticancer effects of ascorbic acid have been investigated in many clinical trials involving different categories of cancer patients. In most of these investigations, high doses of ascorbic acid were mainly used, including during combination therapies with other known anticancer drugs. The results of the investigations in some cancer patient categories were encouraging, and further studies were planned [114,115,116,117,118,119,120,121,122,128,129,130].

The pre-clinical and clinical investigations for the repurposing of ascorbic acid for use as an antioxidant drug in different categories of cancer patients, as well as patients with other diseases, including many diseases associated with ferroptosis, is likely to continue. In most of these cases, ascorbic acid is selected because no other effective treatment with other drugs is available, and also because of safety reasons, since ascorbic acid has low toxicity, even when it is administered at high doses [114,115,116,117,118,119,120,121,122,139,140].

There have been many variations in the clinical protocols involving ascorbic acid, including administration routes and timing, posology, duration of treatment, etc. Furthermore, in most clinical applications or investigations, many possible interactions related to its safety and efficacy parameters have not been considered or monitored. For example, in many cases, the prospects of interactions of ascorbic acid with metal-ion formulations including iron, copper, or zinc, as well as food products containing these metal ions, have not been fully investigated [125,128,129]. Similarly, competition for metal ions and the formation of mixed metal complexes of ascorbic acid may also involve dietary substances and nutraceuticals, such as different polyphenols, many of which also have iron-chelating and or redox-active properties (Figure 3) [26,86,87,141].

The interactions between ascorbic acid and iron may also have implications in the therapeutic activity and toxicity of different drugs that also possess an iron-chelating capacity. For example, the pro-oxidant activity of ascorbic acid with iron in the presence of hydrogen peroxide can be inhibited by L1, DF, mimosine, and maltol, but, in contrast, it can be exacerbated by EDTA [76,142,143]. In this context, several parameters influence the antioxidant activity of ascorbic acid in different clinical conditions, including the presence of iron or other metal ions, and also of drugs and dietary molecules with a metal-chelating capacity (Figure 1, Figure 2 and Figure 3).

Overall, optimized therapeutic protocols of ascorbic acid could be designed based on the evaluation of many of its parameters, including ADMET, pharmacokinetic, and other related characteristics, interactions with iron, and the effects of iron–ascorbate complexes, as well as its interactions with other metal ions, drugs, or dietary molecules with a metal-chelating capacity. Furthermore, several other therapeutic parameters, such as dose protocols and personalized medicine characteristics, may also affect and influence the safety and efficacy of ascorbic acid in various clinical applications and disease conditions.

### 4.2. Efforts for the Development of Quercetin as Clinical Chelator/Antioxidant

Efforts regarding the development of nutraceuticals and many different classes of natural products for use as antioxidant drugs are still continuing in relation to cancer and other diseases, despite difficult dilemmas and setbacks in clinical studies [1,2,3,4,5,6,95]. These observations are similar to those described above in the case of ascorbic acid [95,97,100]. In general, these efforts are based on the main advantages of nutraceuticals and natural products over synthetic drugs, such as their wide accessibility and safety, considering that millions of people, including different categories of patients, are using natural product preparations or nutraceuticals every day as general supplements for health protection [5,7,85].

A major promising class of antioxidants is polyphenols, many of which have been investigated in pre-clinical and clinical settings related to cancer and other diseases [18,25,26,85,87,88,89,90]. Many natural polyphenols are also phytochelators and are known to have metal-binding chelating sites and related properties including, metal-complex formation and redox interactions with iron [19,26,87,88,89,90]. In particular, some polyphenols have being tested in clinical trials with the hope that their antioxidant activity could mainly benefit specific categories of patients that do not respond to established drug therapies [144,145,146,147]. One of these polyphenols is the iron-chelating/antioxidant quercetin, which has shown some promising results in clinical studies and prospects for further development as a pharmaceutical for clinical use (Figure 2) [26,148].

Quercetin is a flavonoid polyphenol mainly found in fruit and vegetables, and it is also used as a food supplement. It is lipophilic (LogP 1.8), sparingly soluble in water with low bioavailability, and it is extensively metabolized in humans [149,150,151,152,153]. It interacts with a variety of metal ions and can form different metal complexes, including iron and copper complexes [154,155,156,157,158,159,160]. Under certain conditions, the presence of copper or iron can accelerate the autoxidation rate of quercetin [161].

Quercetin has been tested in several in vitro, in vivo, and clinical models of different diseases, and has been shown to have mainly antioxidant, anticancer, antimicrobial, and anti-inflammatory activities [160]. Various protocols, including different posology, periods of administration, and other pharmacological parameters, as well as the monitoring of different oxidative stress markers, have been used in clinical studies involving different categories of patients treated with quercetin.

In particular, quercetin has emerged in recent decades as a promising potential therapeutic agent in cardiovascular diseases. Several clinical trials have been carried out examining the different aspects of this category of patients. In one study, quercetin (730 mg/day for 28 days) was shown to reduce the blood pressure significantly in 22 hypertensive persons, but not in 19 pre-hypertensive persons [162]. In another study involving 93 overweight or obese hypertensive persons (25–65 years), quercetin was administered at a dose of 150 mg/day for 42 days. During the treatment, plasma quercetin concentrations increased from 71 to 269 nmol/L, systolic blood pressure and plasma-oxidized LDL concentrations were reduced, while liver and kidney function, hematology, and serum electrolytes did not show any adverse effects [163]. Encouraging results were also observed in clinical studies on myocardial infarction patients. In one study involving 70 patients, the intravenous infusion of quercetin for 5 days in combination with standard therapy was associated with less reperfusion-induced intra-myocardial damage in comparison to the control group of 73 patients treated with standard therapy [164]. However, in another study on post myocardial infarction patients, the administration of quercetin (500 mg/day) for 8 weeks did not affect endothelial dysfunction biomarkers and depression levels [165]. In a recent review, inconsistency in the overall effects of quercetin in cardiovascular diseases was suggested to be due to many factors, including drawbacks related to the clinical trials’ design and the absence of pharmacokinetic/pharmacodynamic tests [166].

Many other clinical trials have also been carried out in different categories of patients, with quercetin administered as an antioxidant adjuvant therapy, which potentially could improve the standard therapy in each condition. Some of these conditions included tissue repair in bone–muscle–tendon regeneration in aging [167], bone loss in postmenopausal women [168,169], polycystic ovary syndrome [170], inflammatory bowel disease [171], diabetes and other metabolic disorders [172], non-alcoholic fatty liver disease [172,173], chronic obstructive pulmonary disease where escalating doses of 500–2000 mg/day of quercetin were used [174], and different forms of toxicity, including those arising from heavy metals, as well as craving in cocaine-dependent patients [175,176].

The interest in the drug development of quercetin increased recently following the first clinical trial using a senolytic combination of quercetin and the antileukemic drug dasatinib, where it was shown that a reduction in senescent cell burden occurred in the adipose tissue of diabetic kidney disease patients and improved physical function occurred in patients with idiopathic pulmonary fibrosis [168,177,178,179]. Both quercetin and dasatinib have metal-binding ligands and capacities, as well as different iron-chelating potentials (Figure 2). In this context, their efficacy, toxicity, and metabolism can be generally affected by the presence of iron or other metal ions, similar to other drugs. A positive outcome related to the use of quercetin was also observed in a preliminary clinical study involving 42 iron-loaded thalassaemia patients who received 500 mg of quercetin per day for 2 weeks, resulting in a significant reduction in serum iron, serum ferritin, and transferrin iron saturation [180].

No serious toxic side-effects have been reported overall in clinical trials involving the above patient groups or patients of various other clinical conditions using quercetin, despite the substantial differences in dose protocols, including the overall wide dose range used (150–2000 mg/day) and the duration of treatment.

Despite the apparent overall safety of quercetin, there is a need for more clinical studies with conclusive findings on its use and the level of therapeutic effects in each clinical condition. Furthermore, more studies and better formulations of quercetin are needed to overcome the many complications associated with low solubility and bioavailability, as well as with the optimization of efficacy in monotherapies and combination therapies with other drugs or nutraceuticals in each targeted clinical condition [148,149,181,182,183,184]. Further studies are also needed on the use of quercetin as an antioxidant chemopreventive adjuvant agent in combination with standard therapies of different diseases, and also as a general chemotherapeutic monotherapy agent. In the meantime, several studies have recently indicated that quercetin can inhibit or modulate ferroptosis, suggesting that quercetin’s iron-binding and antioxidant properties can also lead to the therapeutic targeting of diseases associated with ferroptosis [185,186,187,188,189].

### 4.3. Efforts for the Development of Curcumin as Clinical Chelator/Antioxidant

Curcumin is a polyphenol dietary supplement found mainly in plant products, including turmeric, and is sold as a nutraceutical (Figure 2). There are more than 20,000 publications on the effects of curcumin, including its antioxidant, anti-inflammatory, anticancer, hypoglycemic, antimicrobial, neuroprotective, immunomodulatory, and other biological activities, and also more than 400 clinical trials on different clinical conditions [190,191,192,193].

Beneficial effects regarding its clinical outcomes and/or biomarkers were reported for most studies (75%). These were primarily double-blind, randomized, and placebo-controlled trials where inflammation was the key target, involving obesity and musculoskeletal complications (50%). The next most-studied disease categories were neurological and gastrointestinal disorders, with fewer studies on cancer (9%) [192,194,195]. Similar findings were also reported in different reviews of clinical trial reports, where the antioxidant and anti-inflammatory effects were the main targets identified for the therapeutic action of curcumin [192,196,197].

In general, variable approaches and parameters were selected for the monitoring of curcumin’s efficacy and toxicity in clinical trials involving many different categories of patients. These included different doses (0.5–12.0 g/day), durations of treatment (1 week–22 months), monitoring parameters related to changes in the underlying condition for each category of patients, and also oxidative stress markers or parameters [190,192,194,195,196,197]. Furthermore, other factors influencing these parameters, such as the interactions and effects of drugs, nutraceuticals, dietary constituents, metal ions, etc., on the efficacy and toxicity of curcumin, have not yet been fully investigated or reported [198,199].

Overall, the pharmaceutical prospects and dilemmas for the development of curcumin as an iron-chelating/antioxidant drug are similar to quercetin, where constraints were identified to be due to, among other factors, its hydrophobic molecular nature (LogP 4.16), water insolubility, poor bioavailability, rapid metabolism, systemic elimination, metal-ion interactions, and other interactions [192,200].

In the context of the interaction between curcumin and metal ions, several studies have shown that curcumin binds to heavy and many other metal ions, including iron, boron, cobalt, copper, gallium, gadolinium, gold, lanthanum, manganese, nickel, palladium, platinum, ruthenium, silver, vanadium, and zinc (Figure 2) [201,202,203]. In particular, several studies have suggested the chelation and detoxification of iron and heavy metal ions could lead to the inhibition of heavy-metal-induced lipid peroxidation and iron-induced oxidative damage. It appears that, in general, the binding of toxic metal ions by curcumin is considered to lead to a reduction in the associated metal ion toxicity and also to contribute to its overall antioxidant activity and properties [202,203,204,205].

Several other iron metabolic changes have also been shown in preclinical and clinical studies following the administration of curcumin. In some cases, the prolonged administration of curcumin appears to cause a decrease in iron absorption and iron deficiency [141,206,207]. Similarly, significant decreases in iron absorption and reduction in serum ferritin, transferrin saturation, and serum iron has also been shown in thalassaemia intermedia patients treated with curcumin versus a placebo group [208]. Furthermore, in a double-blind, randomized, controlled clinical trial involving 170 thalassaemia major patients, the administration of curcumin (0.5 g twice daily) for 6 months improved liver function by decreasing liver enzyme levels in comparison to a placebo group [209]. However, serum ferritin or cardiac and liver iron load, which were determined by MRI T2*, were not affected in the same study [209]. In another double-blind, randomized, controlled clinical study on 61 thalassaemia major patients treated with DF, the administration of curcumin (0.5 g twice daily) for 12 weeks caused a significant reduction in serum malondialdehyde and total and direct bilirubin, as well as significantly increased total antioxidant capacity in comparison to the placebo group [210]. Overall, it has been suggested that curcumin administration in combination with DF can improve the antioxidant status in thalassaemia major patients [210].

Further studies are needed to confirm the synergistic effects of curcumin with other drugs in diseases related to iron metabolism, including iron deficiency and iron overload. In addition, several studies have recently suggested that curcumin can induce ferroptosis in different cancer cell types through the incorporation of iron, as well as through other mechanisms [204,211,212,213]. In this context, it appears that the iron-binding and antioxidant properties of curcumin can be used for the therapeutic targeting of diseases associated with ferroptosis (Figure 2) [211,212,213,214,215].

In general, further formulation improvement studies will be needed for increasing the bioavailability of curcumin and its potential development as an antioxidant pharmaceutical for clinical use in diseases associated with FR pathologies and ferroptosis.

### 4.4. Clinical Advances with Maltol, Fisetin and Other Iron Chelating/Antioxidant Phytochelators

There are many other naturally occurring iron-chelating/antioxidants, some of which have been used in pharmaceutical preparations involving formulations with iron and different drugs. In addition to the clinical use of ferrous ascorbate described above, the plant product and food additive maltol, in combination with iron (ferric maltol), has also recently received regulatory approval with the trade name Feraccru or Accrufer for the treatment of iron deficiency [216,217]. After its discovery in 1981, drug development, antioxidant, iron-chelating, and other properties of maltol, and also the properties of its iron- or other metal-ion complexes and their applications, were recently reviewed [216,217,218]. Maltol is a weaker iron chelator and is much more lipophilic (LogP 1.23) than L1 (LogP 0.05), showing potential for development as an iron-chelating/antioxidant pharmaceutical for targeting the lipophilic cellular compartments associated with FR pathologies (Figure 2) [216,217,218]. Similarly, several recent studies have indicated that maltol can be used to inhibit or modulate ferroptosis, suggesting that maltol’s iron-binding, antioxidant, and other properties can lead to the therapeutic targeting of diseases associated with ferroptosis [219,220,221].

Increased interest in the nutraceutical and pharmaceutical development regarding many other categories of phytochelators with iron-chelating/antioxidant properties is also in progress. In particular, the flavonoid fisetin is undergoing clinical trials to be used as a senotherapeutic drug for aging, neurodegenerative, and many other diseases [179,222,223,224,225]. The antioxidant and senolytic properties of fisetin have been shown in many in vitro and in vivo experimental models [226,227,228,229,230]. The iron- and other metal-ion-chelating properties of fisetin have also been previously described in different investigations, suggesting the possible synergistic effect of its antioxidant and iron-chelating properties (Figure 2) [231,232,233,234,235]. Similarly to other iron-chelating molecules, fisetin inhibits ferroptosis and also other oxidative stress processes, including those induced by doxorubicin toxicity [236,237,238,239,240]. The drug development process of fisetin is also in progress, including clinical trials and investigations in different areas regarding its pharmacological and toxicological properties and effects. However, there are also different problems regarding its drug development, including posology, similar to quercetin and curcumin, considering that fisetin also has low solubility and is highly lipophilic (LogP 3.2) [241]. In this context, new fisetin formulations are under investigation in order to increase its solubility and pharmacological activity. Furthermore, it appears that the iron-binding and antioxidant properties of fisetin can be used for the therapeutic targeting of diseases associated with ferroptosis, as shown by recent related investigations on ferroptosis.

Another widely used phytochelator nutraceutical with iron-chelating/antioxidant properties is alpha-lipoic acid (or lipoic acid), which has been studied in many clinical trials in different diseases related to increased oxidative stress, inflammation, and metal intoxication. It has high affinity for iron and other metal ions (Figure 2) [242,243,244,245,246,247]. There are about 100 clinical trials involving lipoic acid, mainly related to diabetic and other neuropathies, as well as cardiomyopathy, kidney disease, and other metabolic diseases. The effects of lipoic acid have also been studied in relation to iron-metabolic disorders, including iron deficiency, iron overload in thalassaemia, and also iron toxicity in Alzheimer’s and other diseases [248,249,250,251,252]. Despite this, some benefits have been shown in sections of patients with the diseases studied, and the overall analysis of the clinical effects are equivocal, suggesting that more doubled-blind randomized clinical trials are required that include improved lipoic acid formulations and therapeutic protocols for targeting specific diseases with FR pathology [242,243,244,245,253]. Preliminary studies have also suggested that lipoic acid can play a role in diseases related to iron- and other metal-ion intoxication, as well as diseases associated, in general, with the modulation of ferroptosis [254,255,256].

There are many other phytochelator and nutraceuticals with iron-chelating/antioxidant properties that require further investigations for clinical development in specific clinical conditions associated with FR pathologies or ferroptosis. These include mimosine, 8-hydroxyquinoline, omadine, emodin, ellagitannins, and silymarin [26].

## 5. Future Prospects for the Clinical Development of Iron-Chelating/Antioxidant Nutraceuticals

No antioxidant drugs including iron chelators/antioxidants have so far been developed or prescribed in medicine for clinical use [5,6]. This paradox is observed despite hundreds of thousands of publications and hundreds of clinical trials supporting the finding that antioxidant activity induced by potential antioxidant drugs or antioxidant nutraceuticals could benefit many categories of patients [1,2,3,4]. In particular, some of the clinical trials in different diseases of FR pathologies have suggested that specific antioxidant nutraceuticals may cause improvements in different OST markers and other pathological parameters [1,2,3,4]. At the same time, millions of people are using antioxidant nutraceuticals and traditional folk medicines every day for chemoprevention or treatment, believing that such natural preparations have beneficial effects on health [7]. Similarly, millions of people are using the metal-chelating drug EDTA in alternative medicine clinics, believing that such interventions have chemopreventive and therapeutic impacts on their life (Figure 1) [139,140,257].

Reluctance toward the development and introduction of antioxidant drugs in medicine by pharmaceutical companies appears to be based mainly on proprietary concerns, high expenditure, and other commercial reasons, including competition and the successful launching of antioxidant compounds and related formulations of nutraceutical products by the nutraceutical industry [5,7]. Another major drawback in antioxidant drug development is the lack of sufficient and robust clinical evidence regarding the effectiveness of antioxidants in clinical tests in different disease categories. In this context, and considering the beneficial effects many patients of different diseases related to FR pathologies may have from the development antioxidant drugs, there is an urgent need for new strategies, including the undertaking of improved and effective co-ordination steps between the various interested parties, including academic establishments and investigators [5]. In the latter case, the new approaches and strategies for the introduction of antioxidant drugs in medicine may include academic groups that are experts on drug-regulatory affairs and translational research, who will undertake, in conjunction with other interested parties, the drug research and development process and the submission of pre-clinical, clinical, and other data to the drug regulatory authorities [5,258,259]. These efforts could be successful, provided that they adhere and fulfill all of the required regulatory procedures for the approval of even one antioxidant drug for specific therapeutic indication (Table 1) [49].

There are several approaches for the design of effective antioxidant therapeutic strategies, including the development of an iron-chelating/antioxidant nutraceutical or natural products for multi-target drug inhibition of Fenton-reaction-related oxidative damage through iron chelation. However, such approaches may be difficult to fully accomplish, considering that, in most cases—including cases involving the iron-chelating drugs L1, DF, and DFRA—there is significant variability in organ distribution and metabolism for each potential antioxidant nutraceutical or natural product, as well as variation in the accessibility of the targets of oxidative damage in different diseases [5,50,51]. For example, out of the three iron-chelating drugs, only L1 could cross the blood–brain barrier and inhibit the oxidative damage caused by iron in the brains of patients with neurodegenerative or other diseases [5,6,50,51].

A further paradox in antioxidant drug development is that improvements in antioxidant biomarker parameters in many clinical studies using investigational antioxidant drugs such as L1, DF, and DFRA, as well as nutraceuticals such as ascorbate, quercetin, and curcumin, so far do not seem to be translated into therapeutic effects or complete therapy in the FR pathology diseases tested [5]. In this context, it is important to note that further investigations are needed to be carried out to clarify the extent of the contribution of the antioxidant effects related to therapeutic outcomes in each disease associated with FR pathologies.

Another parameter for the successful development of potential antioxidant drugs is the identification and targeting of specific diseases associated with FR pathologies where antioxidant drugs could be effective (Table 1). In particular, although academic antioxidant drug-development projects are not common, these may be feasible for orphan or emergency diseases where there are increased mortality and morbidity rate and also where there are no effective treatments available. In the context of iron-chelating drugs, for example, the same procedure for drug development was applied for L1, ferric maltol, and ferrous ascorbate [131,132,136,216,217,218]. In particular, L1 was initially used in iron-loaded patients unable to receive other iron chelation treatment for the removal of excess iron [136,216,217]. Similarly, ferric maltol was also selected for the treatment of iron-deficient inflammatory bowel disease patients because all other iron formulations could not be tolerated [216,217]. Finally, ferrous ascorbate was selected for the treatment of iron-deficient patients in India, mainly because it was less expensive and was also highly effective in treating patients with iron-deficiency-induced anemia in comparison to other drugs [131,132]. Similar approaches in disease selection could be utilized in the drug developmental process of iron-chelating/antioxidant nutraceuticals or other natural products. In such cases, the proposed nutraceutical or other natural product antioxidant could be shown to be therapeutically effective, or at least improve the condition of patients that do not respond to other treatments (Table 1) [5,49].

It is expected that the concerted academic initiatives for nutraceutical or other natural product antioxidant drug development could at least attract generic and small pharmaceutical companies, similarly to the developmental cases of L1 and ferric maltol [72,217,260]. The historical development of L1 is particularly interesting, considering that it was initially registered in India, then Europe, and lastly in the USA for the treatment of iron overload [72,217,260]. Furthermore, the most promising efforts regarding the repurposing of L1 are currently focusing on the treatment of neurodegenerative diseases caused by iron accumulation in the brain or other toxicity with no alternative available therapies [261,262,263,264,265]. It is anticipated that similar efforts and outcomes relating to the development of nutraceuticals and other natural products with iron-chelating antioxidant drugs could also be successful and be used for the treatment of diseases of FR pathologies.

There is wide diversity in the developmental strategies of nutraceuticals and other natural products that are used in iron-chelating antioxidant drugs for clinical use. This generally depends on the target affected by oxidative damage, as well as the pharmacological and other properties of the proposed antioxidant. In this context, the targeting process may include, for example, one or more diseases, different organs, and metabolic or redox pathways. Similarly, other factors may also affect the antioxidant targeting strategy for the selection of a specific antioxidant drug, which, in addition to the mode of action, may also involve other developmental aspects, including, for example, toxicity, posology, and duration of administration, all of which could be critical parameters in each targeting case (Table 1). In this context, the selection of the appropriate nutraceutical or natural product as a potential antioxidant drug for a specific target is likely to be different in each case: for example, it will be different when is considered for chemoprevention compared to when it is considered for short-term administration, such as the treatment of ischemia/reperfusion injury, or long-term administration, such as the treatment of most neurodegenerative diseases [5]. Other aspects of candidate antioxidant drugs that may affect iron-chelating/antioxidant drug strategy include the spectrum of targeting activity, which could range from a single target to a broad spectrum that includes antioxidant drugs targeting various levels, ranging from molecular to tissue to organ targets.

One of the major areas of targeting by potential nutraceutical and other natural product iron-chelating/antioxidant drugs is ferroptosis, a programmed cell death based on the iron catalytic activity damage of cell membrane lipids and also other lipids, which has been identified in almost all diseases and all stages of cancer [36,37,38,39,40,41,42]. Based on this finding, nutraceutical and other natural product iron-chelating/antioxidant drugs could potentially play a major role in the treatment of almost all diseases associated with ferroptosis (Table 1). This prospect further increases the clinical developmental potential of nutraceutical and other natural product iron-chelating antioxidants, not only as monotherapy, but also in combination therapies with other antioxidants and other drugs used in the treatment of other aspects of each specific disease associated with ferroptosis or FR pathologies. Similar combination-therapy approaches were used previously for the treatment of other diseases, including thalassaemia, where iron-chelation combination therapy was, in most cases, more effective than monotherapy [72,84,260].

Future strategies for the development of a new class of antioxidant therapeutics for clinical use may also involve the development of natural (e.g., chlorogenic acid) or synthetic (e.g., aspirin) iron-chelating pro-drugs with antioxidant activity [266,267,268,269]. In particular, it has been suggested that the aspirin iron-chelating metabolites salicylic acid, salicyluric acid, 2,5-dihydroxybenzoic acid (gentisic acid), and 2,3-dihydroxybenzoic acid, which account for more than 70% of the absorbed oral doses of aspirin, may be responsible for the anticancer and iron-deficiency effects observed in long-term, low-dose aspirin users [266,267].

It is anticipated that the antioxidant properties observed and reported in many experimental and clinical studies will be translated through drug research and development process into meaningful therapeutic activity for many diseases with FR pathologies. Similar approaches have been used in other cases in the past through the orphan drug developmental route for other drugs and also investigational drugs originating from academic initiatives such as, for example, the cases of L1 and ferric maltol. It should be noted that the increased prospects of nutraceutical and other natural product iron-chelating/antioxidant drug development may also include combinations with other drugs, such as the combination of fisetin with dasatinib, which could potentially be used for the for enhancement of senolytic activity in aging [230,270].

Overall, it is anticipated that the development of antioxidant drugs, including those with iron-chelating properties, for the inhibition of the Fenton-based oxidative reactions reported in many diseases, as well as many other diseases associated with ferroptosis, will soon become a reality. It is also anticipated that the identification and regulatory approval of any form of antioxidant drug with a specific therapeutic indication could become the start of a new branch of pharmaceuticals and a new era for the use of antioxidant drugs in medicine, including those belonging to the iron-chelating/antioxidant nutraceutical and natural product groups of compounds.

## 6. Conclusions

Substantial scientific and clinical evidence suggests that there is an urgent need for the introduction of antioxidant drugs for clinical use in different conditions involving FR pathologies, including cancer, neurodegenerative, cardiovascular, kidney, liver, and many other diseases. Similarly, increasing scientific evidence suggests that antioxidant drugs, and especially iron chelators/antioxidants, could play a major role in the modulation of ferroptosis, which is also associated with many diseases. Despite the lack of antioxidant drugs, millions of people choose to take daily antioxidant nutraceuticals and other natural products, including iron chelators/antioxidants, and attend alternative medicine clinics for protection against diseases caused by FR toxicity.

Many academic efforts have been undertaken over many years for the clinical testing and development of antioxidant pharmaceuticals including repurposed drugs, nutraceuticals and other natural products in different diseases, but with only limited success so far—for example, in the case of the nutraceuticals ascorbic acid, quercetin, and curcumin.

New academic approaches and strategies are required for the development of antioxidant pharmaceuticals, including efforts for the development of iron-chelating/antioxidant nutraceuticals, within the framework of orphan or emergency drug regulatory requirements, especially in diseases with no proven effective therapies and high morbidity and mortality rates. In this context, the main focus of such strategies should initially be the targeting and treatment of a specific disease, where significant therapeutic improvement in patients without serious toxic side-effects could be shown following randomized clinical trials using the potential antioxidant drug or its combination with other drugs.

There is wide variation in the targeting strategies and development of potential antioxidant drugs for clinical use, ranging from chemoprevention to therapeutic interventions in different diseases. In this context, iron-chelating/antioxidant nutraceuticals and other natural products could, for example, be developed and used for the prevention, or treatment, or post-treatment of oxidative damage and also for long-term, short-term, or intermittent drug administration in each disease associated with FR pathologies. Further strategies could also be designed for identifying synergistic combination therapies between potential antioxidant drug(s), as well as combinations with other drugs, used for the treatment of other aspects of an underlying disease, in addition to oxidative damage. Similarly, new strategies may also involve the identification of pro-drugs such as aspirin, which is biotransformed and partly forms iron-chelating/antioxidant metabolites, and also new formulations, including regulatory-approved nanoparticle preparations for improved therapeutic applications, especially in the case of nutraceuticals with low bioavailability, such as fisetin, quercetin, and curcumin. Most importantly, future strategies could also be designed for the development of iron-chelating/antioxidant nutraceuticals related to the modulation of ferroptosis, a programmed cell-death process identified in almost all stages of cancer and in all other diseases.

It is hoped that the introduction of pharmaceutical antioxidants, including nutraceutical-based iron-chelating/antioxidant drugs and also other forms of antioxidants, will soon become a reality in clinical practice and will aid the treatment of patients with various diseases.

## Figures and Tables

**Table 1:** Summary of properties and developmental efforts of iron-chelating/antioxidant nutraceuticals into pharmaceuticals.

Molecular Weight
Ascorbic acid: 176. Curcumin: 368. Fisetin: 286. Lipoic acid: 206. Maltol: 126. Quercetin: 302.
(Iron-chelating drugs—Deferasirox: 373. Deferiprone: 139. Deferoxamine: 561).
Oil/water partition coefficient (LogP)
Ascorbic acid: 0.01 Curcumin: 4.16. Fisetin: 3.20. Lipoic acid: 2.11. Maltol: 1.23. Quercetin: 1.80.
(Iron-chelating drugs—Deferasirox: 6.30. Deferiprone: 0.05. Deferoxamine: 0.02).
Comparative solubility and bioavailability
Ascorbic acid > Maltol > Quercetin, Lipoic acid, Fisetin, Curcumin.
Main route of administration and posology used in clinical trials
Ascorbic acid: Oral and intravenous (0.20–10.0 g/day). Quercetin: Oral and intravenous (0.15–2.00 g/day). Curcumin: Oral and intravenous (0.50–12.0 g/day). Lipoic acid: Oral and intravenous (0.05–1.80 g/day).
Clinical trials with iron-chelating/antioxidant nutraceuticals
Hundreds of clinical trials on different categories of patients have been carried out, showing improvements in antioxidant markers, but not robust evidence of therapeutic improvements or cures in most cases.
Therapeutic targets related to iron and other metal ion metabolism
Iron binding and inhibition of the Fenton reaction and associated molecular, cellular, and tissue damage in diseases of free radical pathology.
Iron binding and modulation or inhibition of ferroptosis with potential therapeutic implications in associated diseases.
Metal-ion binding other than iron and metal intoxication, including reduction in associated oxidative stress.
Toxicity implications in clinical trials
Low toxicity reported at the range of administered doses.
Strategies for the development of nutraceuticals for clinical use
New formulations and posology protocols are needed for improving the bioavailability and efficacy of the lipophilic nutraceuticals.
There is a need for the identification of specific therapeutic targets in diseases of free radical pathology, which can lead to improvements or treatments of specific categories of patients.
The design of combination therapy protocols with other nutraceuticals, natural compounds, or drugs for the treatment of specific diseases associated with free radical pathology.
Efforts around the development of nutraceuticals for use in pharmaceuticals should adhere to existing legislation and fulfill all of the required regulatory route procedures. This process could be facilitated by involving academic groups that are experts on drug regulatory affairs, as well as government institutions involved in translational research. Similarly, this process could be facilitated by focusing on clinical investigations on orphan or emergency diseases.

## Data Availability

Not applicable.

## Abbreviations

ADMET	absorption, distribution, metabolism, elimination and toxicity
DF	deferoxamine
DFRA	deferasirox
EDTA	ethylenediaminetetraacetic acid
FR	free radicals
iv	intravenous
L1	deferiprone
LVEF	left-ventricular ejection fraction
MRI	magnetic resonance imaging
OST	oxidative stress toxicity
ROS	reactive oxygen species
